# Supplementary material for: A single C-terminal residue controls SARS-CoV-2 spike trafficking and incorporation into VLPs
Source: Nat Commun. 2023 Dec 15;14:8358. doi: 10.1038/s41467-023-44076-3 (PMC10724246; doi:10.1038/s41467-023-44076-3)
Supplement: Supplementary file 1 — Supplementary Information [file 41467_2023_44076_MOESM1_ESM.pdf]

Supplementary Information Inventory

SI Figures

Supplementary Figure S1. Western blot analysis of coatomer pull-down by GST-S tail fusion protein.

Supplementary Figure S2. The binding site in yeast αWD40 (white) and β'WD40 (orange) domains differs in two residues.

Supplementary Figure S3. Similarity in the three S tail-β'WD40 co-crystal structures reported in this investigation.

Supplementary Figure S4. Electron density of critical residues in S tail-WD40 interface highlighted with broken white lines.

Supplementary Figure S5. Conformational change in the basic cluster of αWD40 (white) and β'WD40 (orange) domains.

Supplementary Figure S6. Effect of the Thr1273Glu mutation on the S tail dibasic motif.

Supplementary Figure S7. Solution NMR analysis of S tail peptide binding to WD40 proteins.

SI Tables

Supplementary Table S1. Sequence of S constructs used in this manuscript.

Supplementary Table S2: Identification of coatomer subunits in pull-downs with clientized S tail.

Supplementary Table S3: Crystallographic data collection and refinement statistics.

Supplementary Table S4: Crystallographic data collection and refinement statistics.

Supplementary Table S5. Structural similarity of S tail and previously reported peptides co-crystallized with β'WD40 domain.

Supplementary Table S6: Published interaction affinities of tail peptides for yeast αWD40 and β'WD40 domains.

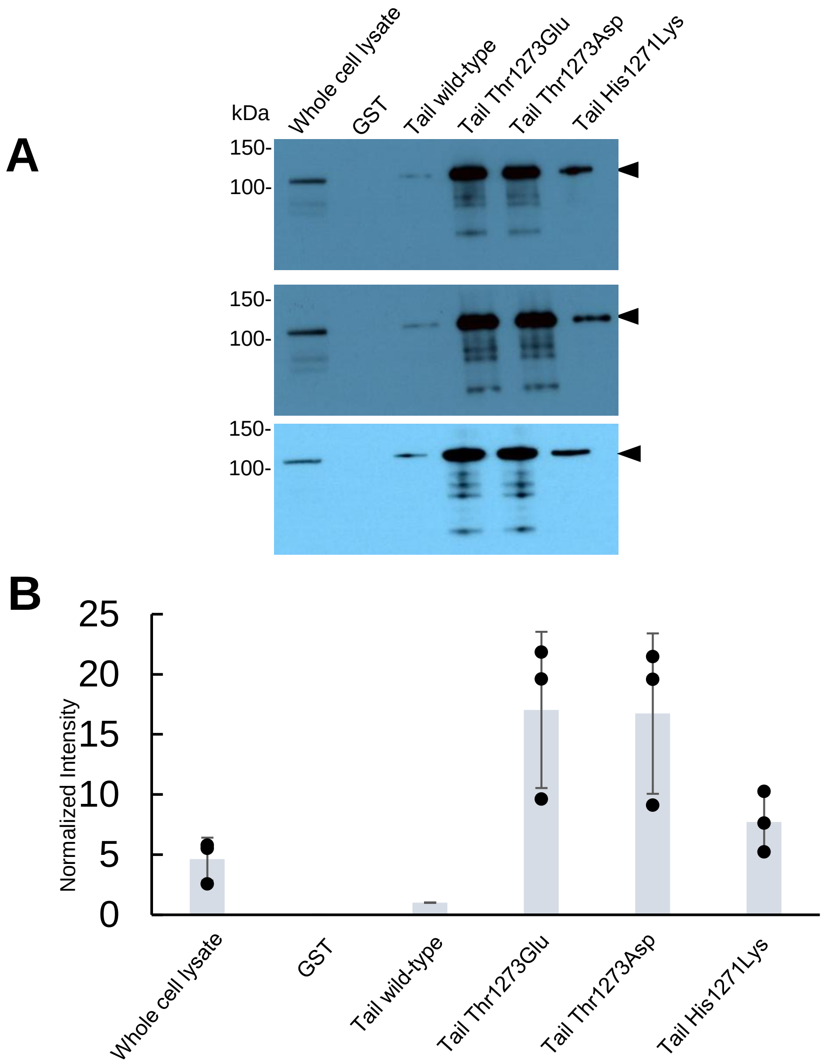

Supplementary Figure S1. Western blot analysis of coatomer pull-down by GST-S tail fusion protein. (A) These panels show three technical replicates of pull-downs probed with an antibody for βCOPI subunit. (B) A bar-graph representation of mean intensity for the β-COPI subunit band. The data have been normalized to the intensity for the wild-type tail pull-down. Individual data points are shown as spheres and data represented as mean of triplicate independent samples ± SD.

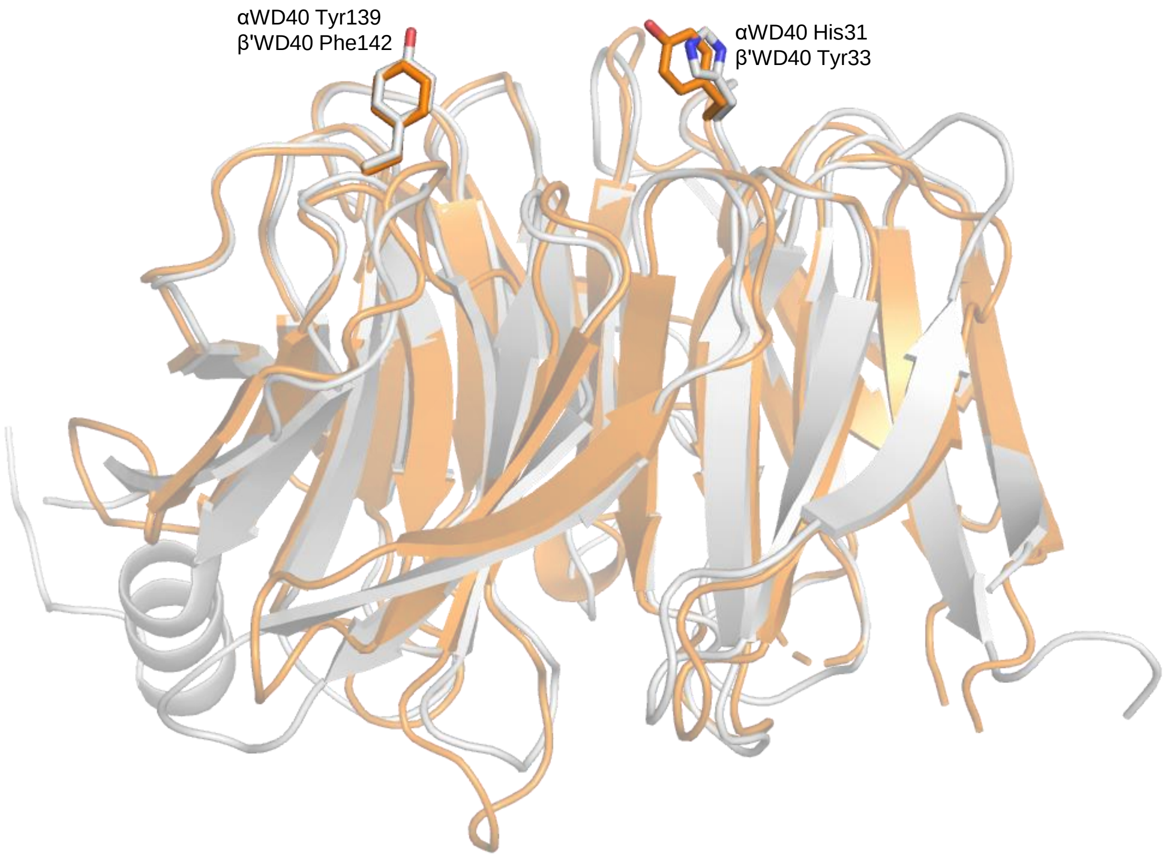

Supplementary Figure S2. The binding site in yeast αWD40 (white) and β'WD40 (orange) domains differs in two residues.

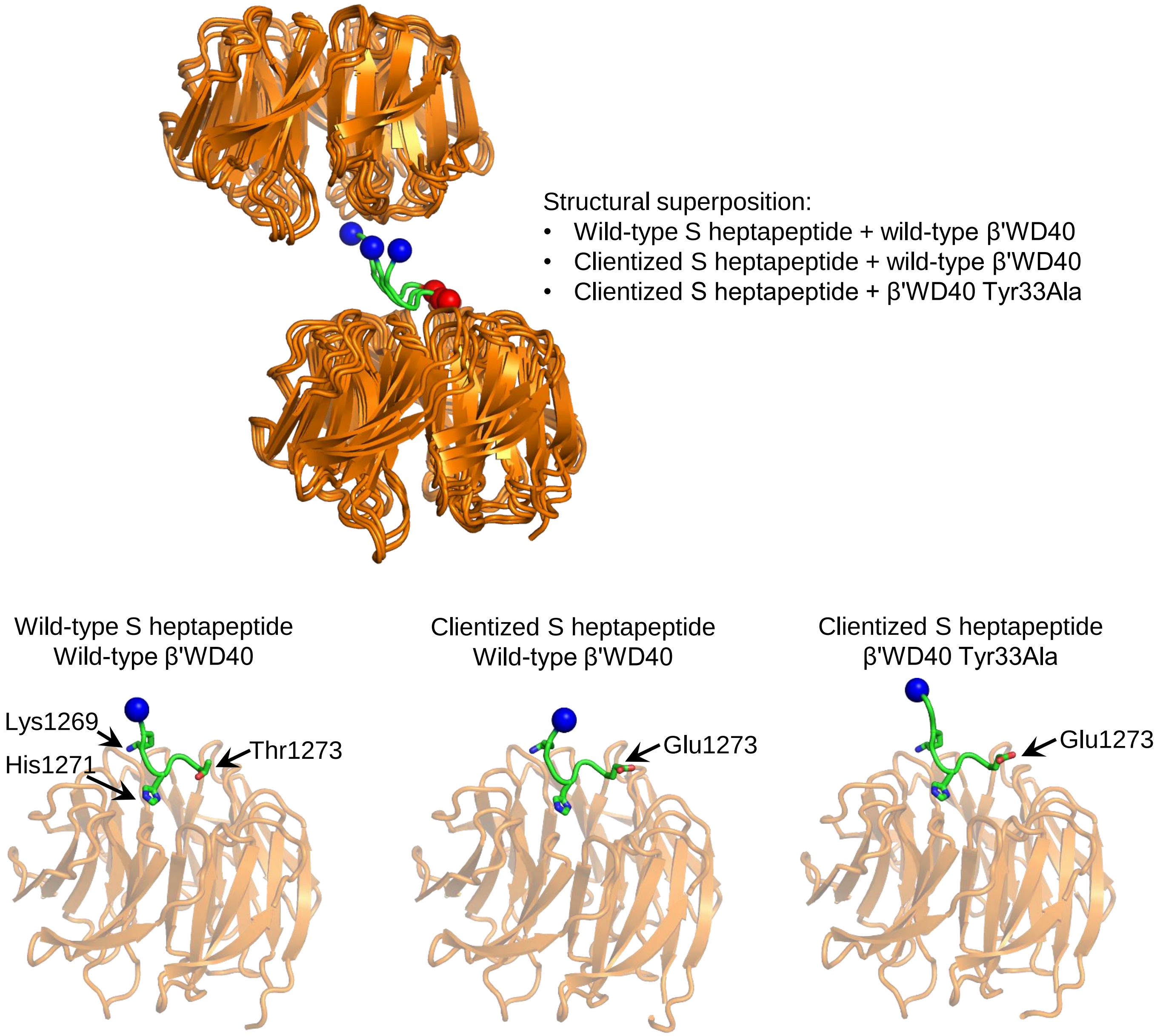

Supplementary Figure S3. Similarity in the three S tail-β'WD40 co-crystal structures reported in this investigation. (Top) Structural superposition of shows similar arrangement of the S tail heptapeptides intercalated between two parallel β'WD40 domains. Blue, red spheres: Cα atoms highlighting the N- and C-termini of the S heptapeptides. (Lower): Individual co-crystal structures of S tail-heptapeptides and β'WD40 domains. The Lys1269, His1271, Thr1273, and Glu1273 side-chains are shown as sticks.

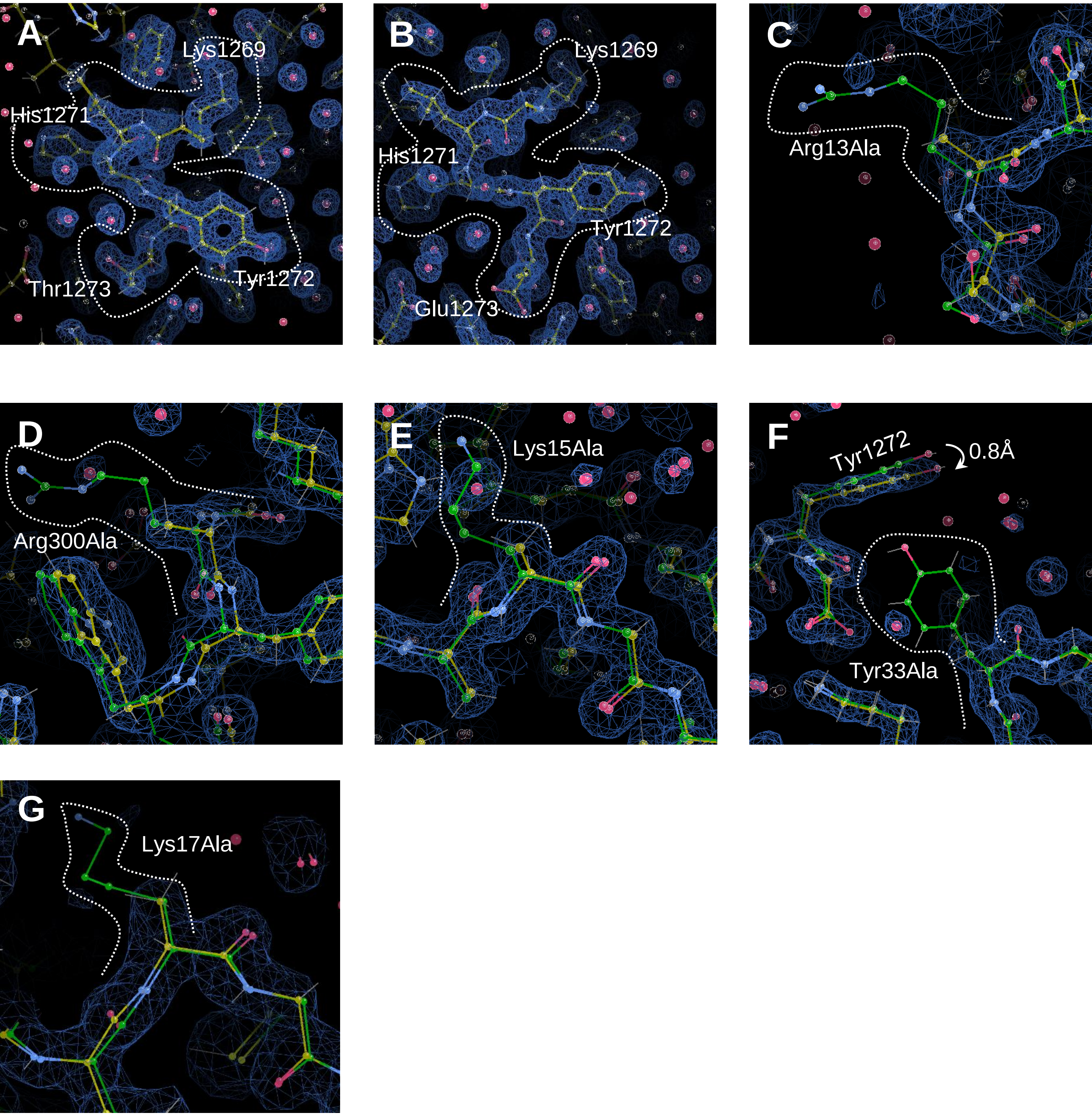

Supplementary Figure S4. Electron density of critical residues in S tail-WD40 interface highlighted with broken white lines. (A) Wild-type S tail hepta-peptide, (B) clientized S tail hepta-peptide, both in β′WD40, and the αWD40 mutation sites at (C) Arg13Ala, (D) Arg300Ala, (E) Lys15Ala, and of β′WD40 at, (G) Lys17Ala. Coordinates corresponding to each electron density map are in yellow whereas superposed wild-type αWD40 in panels (C), (D), (E), and (G) are in green. (F) The co-crystal structure of S tail hepta-peptide with wild-type β'WD40 (green) was superposed onto the co-crystal structure of S tail hepta-peptide with S tail hepta-peptide with β'WD40 Tyr33Ala mutant. The electron density map corresponds to the latter complex. Blue mesh: 2Fo-Fc map, σ=1.2.

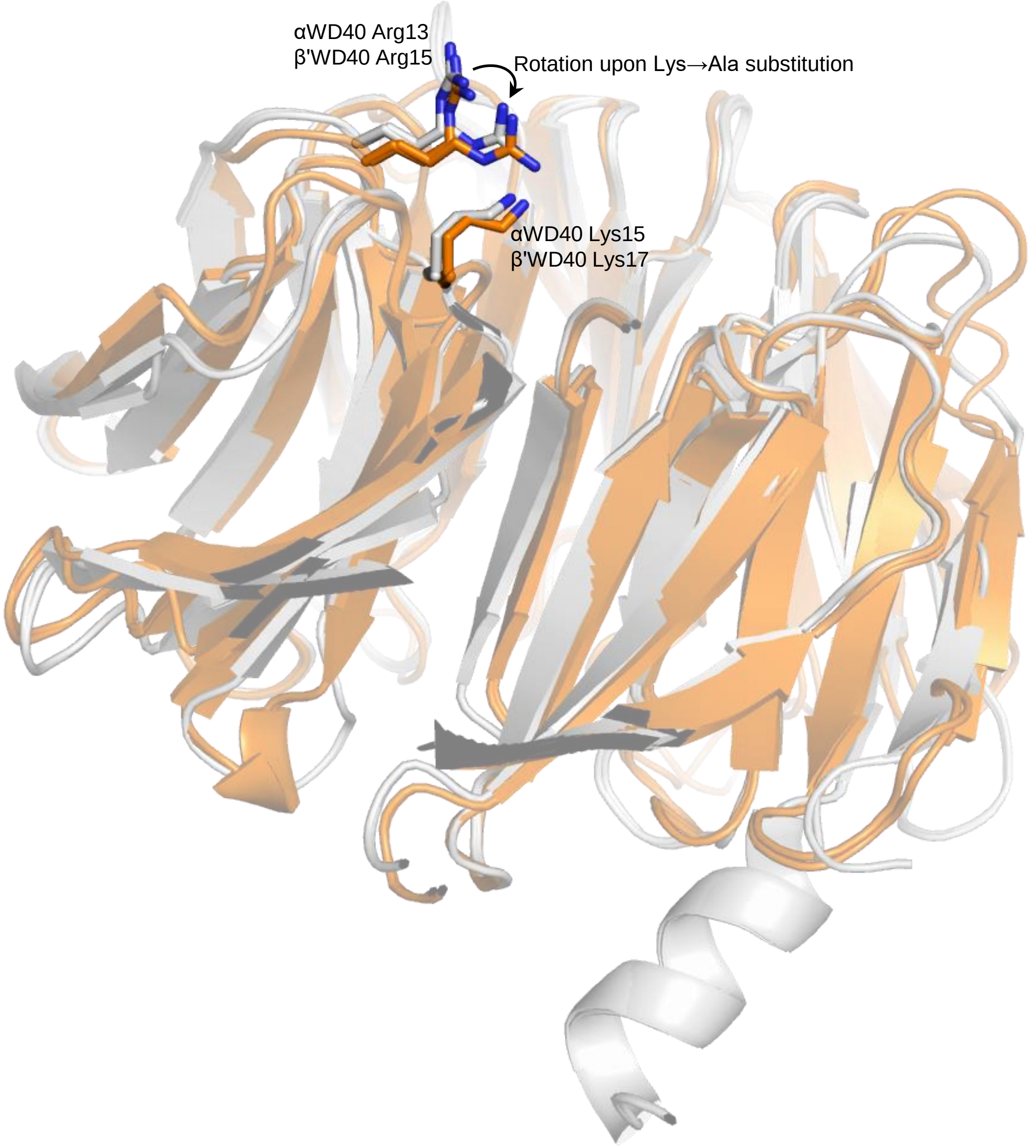

Supplementary Figure S5. Conformational change in the basic cluster of αWD40 (white) and β'WD40 (orange) domains.

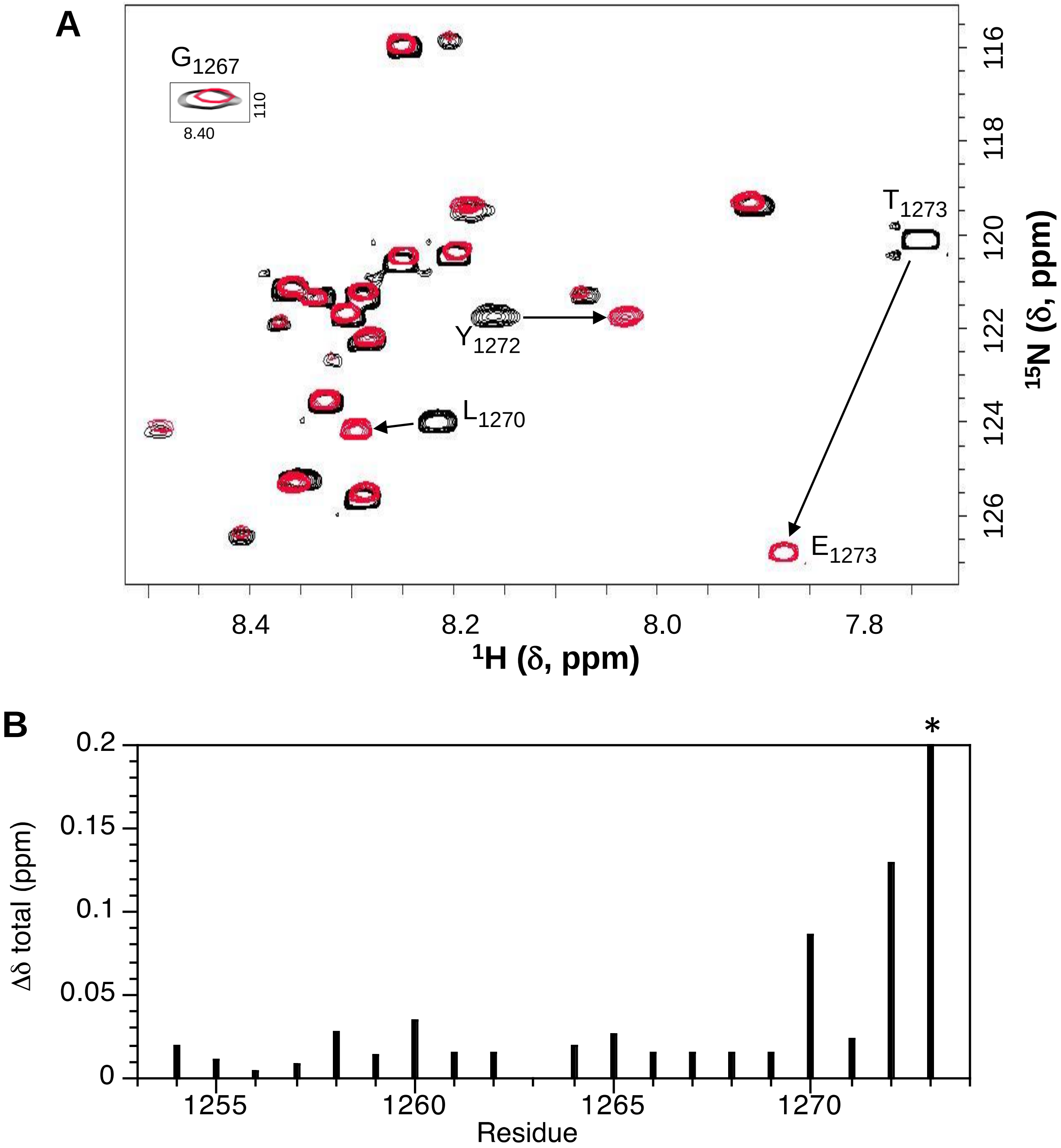

Supplementary Figure S6. Effect of the Thr1273Glu mutation on the S tail dibasic motif. **(A)** Overlaid two-dimensional ^{1}H-^{15}N HSQC spectra of the Cys1253Ala (black) and Cys1253Ala/Thr1273Glu (red) S tail 21-mer peptides. Both spectra were acquired at 25°C in 100 mM potassium phosphate, 0.1 mM DTT, pH 7.0. Peaks with the most notable shifts upon mutation of Thr1273 to Glu1273 are indicated. Residue Gly1267 is shown in the inset. **(B)** Plot of backbone amide chemical shift perturbations between the two spectra shown in panel A. Note that the plot is truncated on the y-axis as the Δδ total value for residue 1273 is 1.34 ppm (asterisk).

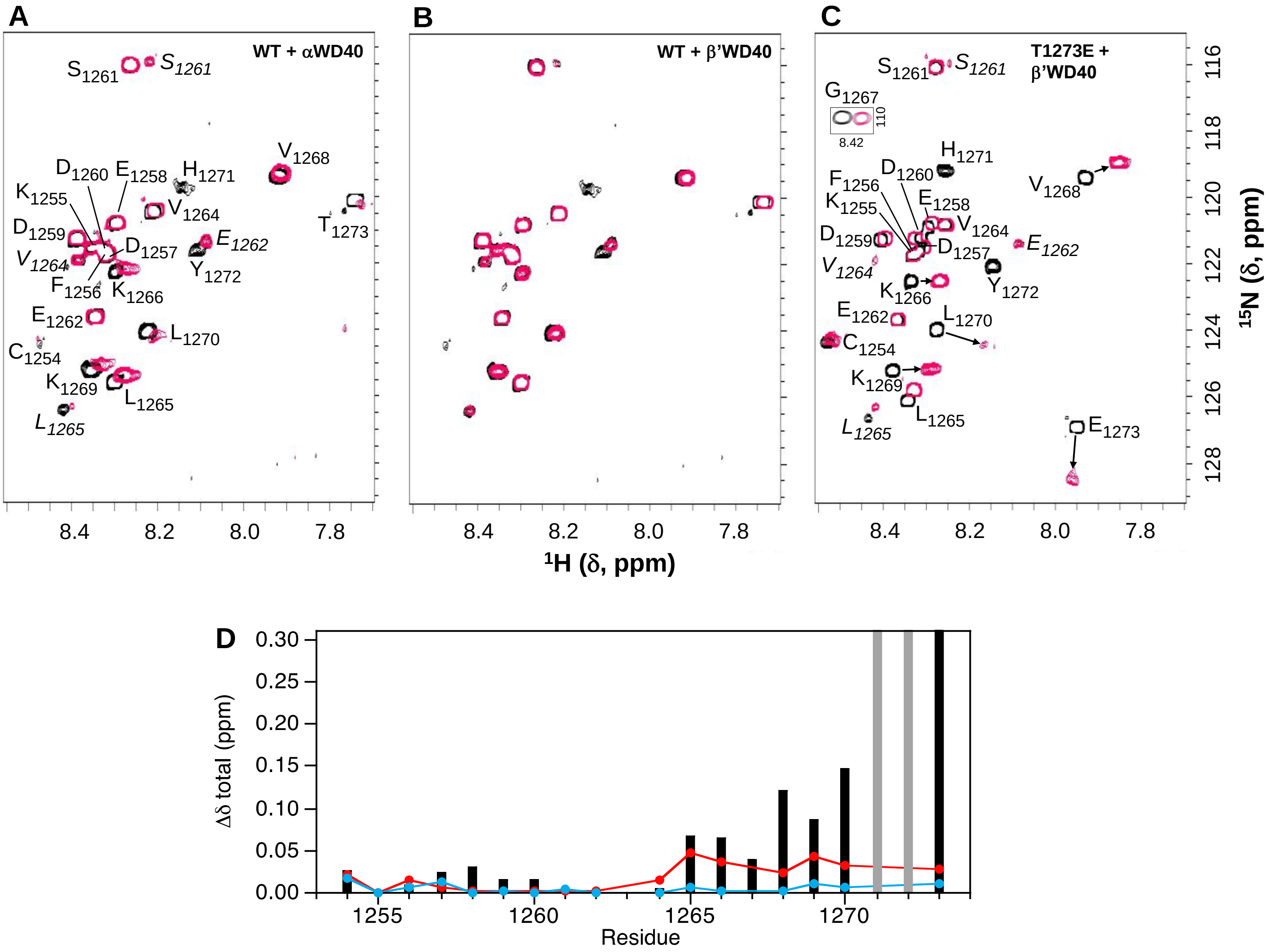

Supplementary Figure S7. Solution NMR analysis of S tail peptide binding to WD40 proteins. **(A)** Two dimensional ^1H-^{15}N HSQC spectra of wild-type S tail peptide in free (black) and aWD40-bound (red) states. Backbone amide assignments are as described in Figure 4. **(B)** Two dimensional ^1H-^{15}N HSQC spectra of wild-type S tail peptide in free (black) and β'WD40-bound (red) states. **(C)** Two dimensional ^1H-^{15}N HSQC spectra of Cys1253Ala-Thr1273Glu S tail peptide in free (black) and β'WD40-bound (red) states. Assignments in the bound state are indicated and were made based on patterns observed in panel A. Residue Gly1267 is exchange broadened in the free form of wild-type S tail but is detected in the mutant S tail (inset box). **(D)** Chemical shift perturbation plots for the data in panels A (red), B (blue), and C (black). The gray bars indicate exchange broadening of His1271 and Tyr1272 upon addition of WD40 proteins in all three binding experiments.

Supplementary Table S1. Sequence of S constructs used in this manuscript[1]

Assay	Protein/peptide	Sequence
**Immuno-fluorescence assays**	Wild-type	Full-length, 1273 residue
	Thr1273Glu clientized	Full-length, 1273 residue
**Coatomer pull-down**	Wild-type	GST- LKGCCSCGSCCKFDEDDSEPVLKGVKLHYT
	His1271Lys mutant	GST- LKGCCSCGSCCKFDEDDSEPVLKGVKL**K**YT
	Lys1269Ala/His1271Ala mutant	GST- LKGCCSCGSCCKFDEDDSEPVLKGV**A**L**A**YT
	Thr1273Glu clientized	GST- LKGCCSCGSCCKFDEDDSEPVLKGVKLHY**E**
	Thr1273Asp clientized	GST- LKGCCSCGSCCKFDEDDSEPVLKGVKLHY**D**
**BLI**	Wild-type heptapeptide	Biotin-PEG_linker- GVKLHYT
	Tyr1272Ala mutant heptapeptide	Biotin-PEG_linker- GVKLH**A**T
	Clientized heptapeptide	Biotin-PEG_linker- GVKLHY**E**
	Scrambled clientized heptapeptide	Biotin-PEG_linker- K**E**VYLHG
	Wild-type 21mer peptide	Biotin- CCKFDEDDSEPVLKGVKLHYT
	Clientized 21mer peptide	Biotin- CCKFDEDDSEPVLKGVKLHY**E**
**HSQC**	Wild-type 21mer peptide	CCKFDEDDSEPVLKGVKLHYT
	Clientized 21mer peptide (Cys1253Ala)	ACKFDEDDSEPVLKGVKLHY**E**
**PRE**	Wild-type 21mer peptide (Cys1253Ala)	ACKFDEDDSEPVLKGVKLHYT
	Clientized 21mer peptide (Cys1253Ala)	ACKFDEDDSEPVLKGVKLHY**E**
**ACE2 binding and VLP assays**	Wild-type	Full-length, 1273 residue
	Thr1273Asp clientized	Full-length, 1273 residue
	Thr1273Glu clientized	Full-length, 1273 residue

[1]Residue numbering (UNIPROT ID P0DTC2):

^{1244}LKGCCSC^{1250} ^{1251}GSCCKFDEDD^{1260} ^{1261}SEPVLKGVKLHYT^{1273}

Supplementary Table S2. Identification of coatomer subunits in pull-downs with clientized S tail

Accession	Gene Symbol	Description	Sum PEP Score	Sequence Coverage [%]	# Peptides	Abundance: Thr1273Asp	Abundance: Thr1273Glu
Q8CIE6	Copa	Coatomer subunit α	126.501	23	22	High	High
Q9JIF7	Copb1	Coatomer subunit β	36.418	10	7	High	High
O55029	Copb2	Coatomer subunit β'	69.231	20	13	High	High
Q5XJY5	Arcn1	Coatomer subunit δ	1.588	2	1	Peak Found	High
O89079	Cope	Coatomer subunit ε	31.878	34	7	High	High
Q9QZE5	Copg1	Coatomer subunit γ-1	12.841	4	3	High	High
P61924	Copz1	Coatomer subunit ζ-1	18.131	21	2	High	High

Supplementary Table S3. Crystallographic data collection and refinement statistics.

**Protein**	β'WD40	β'WD40	β'WD40-Tyr33Ala
S peptide	GVKLHYT	GVKLHYE	GVKLHYE
PDB ID	8ENS	8ENW	8ENX
**Data collection**[1]			
Space group	C121	P1	P1
Unit cell (a, b, c; Å)	75.0, 51.4, 85.3	42.7, 46.0, 84.3	42.8, 46.2, 84.5
Unit cell (α, β, γ; °)	90.0, 101.7, 90.0	81.2, 81.5, 69.0	81.1, 81.7, 69.5
Resolution (Å)	28.1-1.4 (1.47-1.45)	82.9-1.4 (1.47-1.45)	28.3-1.8 (1.84-1.80)
R_{sym} (%)	10.7 (67.1)	9.6 (88.4)	13.7 (71.3)
<I/σ(I)>	9.1 (2.3)	6.8 (1.4)	3.7 (1.0)
CC1/2	1.00 (0.84)	1.00 (0.62)	0.99 (0.55)
Redundancy	6.9 (6.3)	3.7 (3.8)	2.4 (2.4)
Total number of reflections	390433 (17128)	370084 (18804)	128752 (7007)
Number of unique reflections	56398 (2705)	100965 (4966)	53029 (2959)
Completeness (%)	99.8 (96.4)	96.6 (94.8)	96.6 (90.2)
Wilson B factor (Å^2)	14.4	15.2	16.1
**Refinement**			
Resolution used in refinement (Å)	28.1-1.4 (1.47-1.45)	42.7-1.4 (1.47-1.45)	28.3-1.8 (1.84-1.80)
Number of WD40 chains in ASU	1	2	2
Number of spike peptide chains in ASU	1	2	1
Number of reflections used in refinement	56384 (2736)	100938 (3284)	53018 (5144)
R_{work} (%)	15.1 (22.5)	15.3 (25.6)	14.9 (24.4)
R_{free} (%)[2]	17.2 (24.0)	18.0 (29.8)	19.6 (28.8)
Amino acid residues in final model	308	617	611
Atoms in final model	5307	10385	10502
Water molecules	469	530	826
Hydrogen atoms	2355	4839	4774
All atom clash-score	1.6	1.8	2.0
Ramachandran angles (favored, %)	94.7	96.6	95.0
Ramachandran angles (allowed, %)	5.3	4.4	5.0
Ramachandran angles (outliers, %)	0.0	0.0	0.0
Rotamers (outliers, %)	0.7	0.0	0.0
Cβ outliers	0	0	0
RMS bond length (Å)	0.008	0.005	0.005
RMS bond angle (°)	1.086	0.946	0.759

[1]Data collected at NSLSII 17-ID-1 AMX beamline at an X-ray wavelength of 0.92Å. [2]5% of reflections assigned for R_{free} calculations.

Supplementary Table S4. Crystallographic data collection and refinement statistics.

**Protein**	αWD40-Arg13Ala	αWD40-Lys15Ala	αWD40-Arg300Ala	β'WD40-Lys17Ala
PDB ID	8ENY	8ENZ	8EO0	8SZX
**Data collection**[1]				
Space group	P1211	P1211	P1211	I41
Unit cell (a, b, c; Å)	37.4, 170.2, 71.5	37.5, 172.3, 71.5	37.9, 171.3, 71.6	158.2, 158.2, 47.6
Unit cell (α, β, γ; °)	90.0, 99.8, 90.0	90.1, 99.9, 90.0	89.9, 100.3, 90.1	90.1, 90.0, 90.1
Resolution (Å)	28.8-1.9 (1.94-1.90)	28.8-1.6 (1.68-1.65)	28.9-1.8 (1.83-1.80)	27.9-2.0 (2.05-2.00)
R_{sym} (%)	12.9 (84.5)	7.0 (44.5)	18.5 (19.6)	24.3 (65.7)
$<I/\sigma(I)>$	5.9 (1.4)	8.4 (2.2)	4.6 (1.1)	3.9 (1.8)
CC1/2	0.99(0.57)	0.99 (0.80)	0.99 (0.55)	0.95 (0.68)
Redundancy	4.8 (4.4)	3.6 (3.7)	4.7 (4.7)	4.7 (4.7)
Total number of reflections	328403 (20135)	381341 (19084)	391365 (21430)	186781 (13859)
Number of unique reflections	67929 (4533)	105242 (5174)	82865 (4564)	40138 (2931)
Completeness (%)	98.2 (97.1)	98.6 (98.2)	100.0 (99.9)	99.9 (100.0)
Wilson B factor ($Å^2$)	23.9	17.7	20.2	16.1
**Refinement**				
Resolution used in refinement (Å)	28.4 - 1.9 (1.96 - 1.90)	28.7 - 1.6 (1.71 - 1.65)	28.9 - 1.8 (1.86 - 1.80)	27.9 - 2.0 (2.07 - 2.00)
Number of WD40 chains in ASU	3	3	3	2
Number of reflections used in refinement	67828 (6755)	105146 (10362)	82725 (8247)	40131 (4001)
R_{work} (%)	17.8 (25.1)	16.3 (24.8)	17.7 (28.3)	19.0 (24.8)
R_{free} (%)[2]	21.9 (29.4)	19.3 (26.4)	21.8 (32.7)	23.3 (28.9)
Amino acid residues in final model	917	917	915	603
Atoms in final model	15162	15450	15177	10037
Water molecules	453	735	525	464
Hydrogen atoms	7234	7234	7203	4721
All atom clash-score	2.9	2.9	2.7	2.2
Ramachandran angles (favored, %)	95.4	95.5	95.7	95.2
Ramachandran angles (allowed, %)	4.6	4.5	4.3	4.8
Ramachandran angles (outliers, %)	0.0	0.0	0.0	0.0
Rotamers (outliers, %)	0.2	0.7	0.4	0.5
Cβ outliers	0.0	0.0	0.0	0.0
RMS bond length (Å)	0.002	0.010	0.004	0.002
RMS bond angle (°)	0.600	1.030	0.770	0.620

[1]Data collected at NSLSII 17-ID-1 AMX beamline at an X-ray wavelength of 0.92Å. [2]5% of reflections assigned for R_{free} calculations.

Supplementary Table S5. Structural similarity of S tail and previously reported peptides co-crystallized with β'WD40 domain[1].

PDB ID	Peptide source	Peptide sequence	Cα RMSD (Å) (GVKLHYT)	Peptide footprint on β'WD40
2YNN[2]	--	KTKTN	1.0	Arg15, Lys17, Tyr33, Arg59, Arg101, Met144, Asn188, Asp206, Arg272, Trp274
4J77[3]	hWbp1	EKEKSD	0.3	Arg15, Lys17, Tyr33, Arg59, Asp98, Tyr99, Arg101, Asp117, His141, Phe142, Met144, Leu161, Asn188, Asp206, Arg272, Trp274
4J78[3]	Emp47p	IKTKLL	0.1	Arg15, Lys17, Tyr33, Arg59, Asp98, Tyr99, Arg101, Asp117, His141, Phe142, Met144, Leu161, Asn188, Asp206, Arg272, Trp274
4J79[3]	PEDV spike	FEKVHVQ	0.5	Arg15, Lys17, Tyr33, Arg59, Asp98, Tyr99<br>Arg101, Asp117, Phe142, Met144, Leu161<br>Asn188, Asp206, Arg272
4J81[3]	Insig-1	PEKPHSD	0.3	Arg15, Lys17, Tyr33, Arg59, Asp98, Tyr99, Arg101, Asp117, Phe142, Met144, Asn188, Asp206, Arg272
4J82[3]	Insig-2	AEKSHQE	0.2	Arg15, Lys17, Tyr33, Arg59, Asp98, Tyr99, Arg101, Asp117, Phe142, Met144, Leu161, Asn188, Asp206, Arg272

[1]Alignment performed over five C-terminal residues of peptide only.
[2,3]References:

[2]Jackson, L. P. et al. Molecular basis for recognition of dilysine trafficking motifs by COPI. Dev Cell 23, 1255-1262, doi:10.1016/j.devcel.2012.10.017 (2012).
[3]Ma, W. & Goldberg, J. Rules for the recognition of dilysine retrieval motifs by coatomer. EMBO J 32, 926-937, doi:10.1038/emboj.2013.41 (2013).

Color code: Basic cluster residues, other shared contact residues within 4Å

Supplementary Table S6. Published interaction affinities of tail peptides for yeast αWD40 and β' WD40 domains

Client	Peptide Sequence	K_D (αWD40, μM)	K_D (β'WD40, μM)
SARS-CoV-2 spike[1]	G-V-K-L-H-Y-T	1.40 ± 0.15	n.d.
SARS-CoV-2 spike Thr1273Glu[1]	G-V-K-L-H-Y-E	0.31 ± 0.01	9.1 ± 0.83
p25[2]	F-E-A-K-K-L-V	11.3 ± 0.6	27.7 ± 2.5
E19[2]	R-R-S-F-I-D-E-K-K-M-P	64.5 ± 5.4	49.0 ± 2.2
yWbp1[2]	T-F-K-K-T-N	36.6 ± 1.5	170.9 ± 10.2
hWbp1[2]	E-K-E-K-S-D	2.9 ± 0.1	3.4 ± 0.3
Emp47p[2]	I-K-T-K-L-L	16.1 ± 0.6	22.5 ± 1.3
Scyl1[2]	G-A-R-K-L-D		20.4 ± 0.7
PEDV-spike[2]	F-E-K-V-H-V-Q	81.9 ± 11.0	>800.0
Insig-1[2]	P-E-K-P-H-S-D	27.9 ± 2.6	31.9 ± 2.6
Insig-2[2]	A-E-K-S-H-Q-E	22.7 ± 1.4	44.2 ± 1.6
KKTN motif[3]	C-T-F-K-K-T-N	--	85.0
Emp47p[3]	R-Q-E-I-I-K-T-K-L-L	--	6.8 ± 2.6

[1]Dey D, Singh S, Khan S, Martin M, Schnicker NJ, Gakhar L, Pierce BG, Hasan SS. An extended motif in the SARS-CoV-2 spike modulates binding and release of host coatomer in retrograde trafficking. Commun Biol. 2022 Feb 8;5(1):115. doi: 10.1038/s42003-022-03063-y.
[2]Ma, W. & Goldberg, J. Rules for the recognition of dilysine retrieval motifs by coatomer. EMBO J 32, 926-937, doi:10.1038/emboj.2013.41 (2013).
[3]Jackson, L. P. et al. Molecular basis for recognition of dilysine trafficking motifs by COPI. Dev Cell 23, 1255-1262, doi:10.1016/j.devcel.2012.10.017 (2012).
