# Supplementary material for: A single C-terminal residue controls SARS-CoV-2 spike trafficking and incorporation into VLPs
Source: Nat Commun. 2023 Dec 15;14:8358. doi: 10.1038/s41467-023-44076-3 (PMC10724246; doi:10.1038/s41467-023-44076-3)
Supplement: Supplementary file 3 — Reporting Summary [file 41467_2023_44076_MOESM3_ESM.pdf]

nature portfolio

Corresponding author(s): S SAIF HASAN

Last updated by author(s): Nov 26, 2023

Reporting Summary

Nature Portfolio wishes to improve the reproducibility of the work that we publish. This form provides structure for consistency and transparency in reporting. For further information on Nature Portfolio policies, see our Editorial Policies and the Editorial Policy Checklist.

Statistics

For all statistical analyses, confirm that the following items are present in the figure legend, table legend, main text, or Methods section.

n/a	Confirmed	
☐	☒	The exact sample size (*n*) for each experimental group/condition, given as a discrete number and unit of measurement
☐	☒	A statement on whether measurements were taken from distinct samples or whether the same sample was measured repeatedly
☒	☐	The statistical test(s) used AND whether they are one- or two-sided<br>*Only common tests should be described solely by name; describe more complex techniques in the Methods section.*
☒	☐	A description of all covariates tested
☒	☐	A description of any assumptions or corrections, such as tests of normality and adjustment for multiple comparisons
☐	☒	A full description of the statistical parameters including central tendency (e.g. means) or other basic estimates (e.g. regression coefficient) AND variation (e.g. standard deviation) or associated estimates of uncertainty (e.g. confidence intervals)
☒	☐	For null hypothesis testing, the test statistic (e.g. *F*, *t*, *r*) with confidence intervals, effect sizes, degrees of freedom and *P* value noted<br>*Give P values as exact values whenever suitable.*
☒	☐	For Bayesian analysis, information on the choice of priors and Markov chain Monte Carlo settings
☒	☐	For hierarchical and complex designs, identification of the appropriate level for tests and full reporting of outcomes
☒	☐	Estimates of effect sizes (e.g. Cohen's *d*, Pearson's *r*), indicating how they were calculated

Our web collection on statistics for biologists contains articles on many of the points above.

Software and code

Policy information about availability of computer code

Data collection	ForteBio Suite, LSDC
Data analysis	ForteBio Suite, XDS, CCP4, Phenix, Coot, PyMol, ImageJ, NMRPIPE, Sparky, Proteome Discoverer

For manuscripts utilizing custom algorithms or software that are central to the research but not yet described in published literature, software must be made available to editors and reviewers. We strongly encourage code deposition in a community repository (e.g. GitHub). See the Nature Portfolio guidelines for submitting code & software for further information.

Data

Policy information about availability of data

All manuscripts must include a data availability statement. This statement should provide the following information, where applicable:

- Accession codes, unique identifiers, or web links for publicly available datasets
- A description of any restrictions on data availability
- For clinical datasets or third party data, please ensure that the statement adheres to our policy

Source data are published as an accompanying file. All plasmids will be available through Addgene. All crystal structures have been deposited in the Protein Data Bank (PDB) with accession IDs: 8ENS (https://www.rcsb.org/structure/8ENS; β'WD40 with wild-type S tail heptapeptide), 8ENW (https://www.rcsb.org/structure/8ENW; β'WD40 with Thr1273Glu clientized S tail heptapeptide), 8ENX (https://www.rcsb.org/structure/8ENX; β'WD40-Tyr33Ala with Thr1273Glu clientized S tail heptapeptide), 8ENY (https://www.rcsb.org/structure/8ENY; αWD40-Arg13Ala), 8ENZ (https://www.rcsb.org/structure/8ENZ; αWD40-Lys15Ala), 8EO0 (https://www.rcsb.org/structure/8EO0; αWD40-Arg300Ala), and 8SZX (https://www.rcsb.org/structure/8SZX; β'WD40-Lys17Ala). NMR assignments for the

wild-type and mutant S tails have been deposited in the BioMagResBank (BMRB) with accession codes 51663 (doi:10.13018/BMR51663) and 52007 (doi:10.13018/BMR52007), respectively.

Research involving human participants, their data, or biological material

Policy information about studies with human participants or human data. See also policy information about sex, gender (identity/presentation), and sexual orientation and race, ethnicity and racism.

Reporting on sex and gender	No human participation; no live animal experiments; sex, gender, sexual orientation, race, ethnicity, racism not applicable
Reporting on race, ethnicity, or other socially relevant groupings	No human participation; no live animal experiments; sex, gender, sexual orientation, race, ethnicity, racism not applicable
Population characteristics	No human participation and no population characterization; no live animal experiments; sex, gender, sexual orientation, race, ethnicity, racism not applicable
Recruitment	No human participation; no recruitment of human participants
Ethics oversight	No human participation; ethics not applicablee

Note that full information on the approval of the study protocol must also be provided in the manuscript.

Field-specific reporting

Please select the one below that is the best fit for your research. If you are not sure, read the appropriate sections before making your selection.

☒ Life sciences ☐ Behavioural & social sciences ☐ Ecological, evolutionary & environmental sciences

For a reference copy of the document with all sections, see nature.com/documents/nr-reporting-summary-flat.pdf

Life sciences study design

All studies must disclose on these points even when the disclosure is negative.

Sample size	No pre-detrmination of sample size was performed
Data exclusions	5% of reflections were excluded to calculate R-free in crystallographic data analysis. This is mentioned in SI Tables S3 and S4.
Replication	Replicates were successful.
Randomization	No randomization was performed.
Blinding	No blinding was performed.

Reporting for specific materials, systems and methods

We require information from authors about some types of materials, experimental systems and methods used in many studies. Here, indicate whether each material, system or method listed is relevant to your study. If you are not sure if a list item applies to your research, read the appropriate section before selecting a response.

Materials & experimental systems

n/a	Involved in the study	
☐	☒	Antibodies
☐	☒	Eukaryotic cell lines
☒	☐	Palaeontology and archaeology
☒	☐	Animals and other organisms
☒	☐	Clinical data
☒	☐	Dual use research of concern
☒	☐	Plants

Methods

n/a	Involved in the study	
☒	☐	ChIP-seq
☒	☐	Flow cytometry
☒	☐	MRI-based neuroimaging

Antibodies

Antibodies used	Antibodies. The following antibodies were used in this investigation: SARS-CoV/SARS-CoV-2 S monoclonal antibody 1A9 (Cat. No. GTX632604; GeneTex, USA; 1:1000 dilution); goat anti-mouse IgG (H+L) highly cross-adsorbed secondary antibody, Alexa Fluor™ Plus 488 (Cat. No. A-11001; Invitrogen, USA; 1:700 dilution); goat anti-rabbit IgG (H+L) highly cross-adsorbed secondary antibody, Alexa

	Fluor™ 555 (Cat. No. A32732; Invitrogen, USA; 1:700 dilution); SARS-CoV-2 (2019-nCoV) S antibody, rabbit polyclonal antibody, antigen affinity purified (Cat. No. 40591-T62; Sino Biological, USA; 1:1000 dilution); SARS-CoV/SARS-CoV-2 S monoclonal antibody 1A9 (Cat. No. MA5-35946; Thermo Fisher Scientific, USA; 1:1000 dilution); goat anti-human IgG-HRP (Cat. No. sc-2907; Santa Cruz Biotechnology Inc, USA; 1:5000 dilution); ACE2 recombinant rabbit monoclonal antibody (SN0754) (Cat. No. MA5-32307; Thermo Fisher Scientific, USA; 1:1000 dilution); mouse anti-β-actin-peroxide monoclonal antibody (Cat. No. A3854; Sigma-Aldrich, USA; 1:10,000 dilution); rabbit anti-β-COPI polyclonal antibody (Cat. No. ab2899; Abcam, USA; 1:1000 dilution); rabbit anti-giantin polyclonal antibody (Cat. No. 924302; BioLegend, USA; 1:1000 dilution).
Validation	All antibodies were commercially sourced.

Eukaryotic cell lines

Policy information about cell lines and Sex and Gender in Research

Cell line source(s)	The eukaryotic cell lines used in this manuscript are as follows: Expi293F (Cat. No. A14527, Thermo Fisher Scientific, USA), HEK293T (Cat. No. CRL-3216, ATCC), and HeLa (Cat. No. CCL-1, ATCC).
Authentication	All cell lines were commercially sourced.
Mycoplasma contamination	Not checked.
Commonly misidentified lines (See ICLAC register)	Not applicable.

Plants

Seed stocks	No plants used in this investigation.
Novel plant genotypes	No plants used in this investigation.
Authentication	No plants used in this investigation.
