# Supplementary material for: A single C-terminal residue controls SARS-CoV-2 spike trafficking and incorporation into VLPs
Source: Nat Commun. 2023 Dec 15;14:8358. doi: 10.1038/s41467-023-44076-3 (PMC10724246; doi:10.1038/s41467-023-44076-3)
Supplement: Supplementary file 4 — Source Data [file 41467_2023_44076_MOESM4_ESM.zip › Uncropped blots.PPT]

## Slide 1
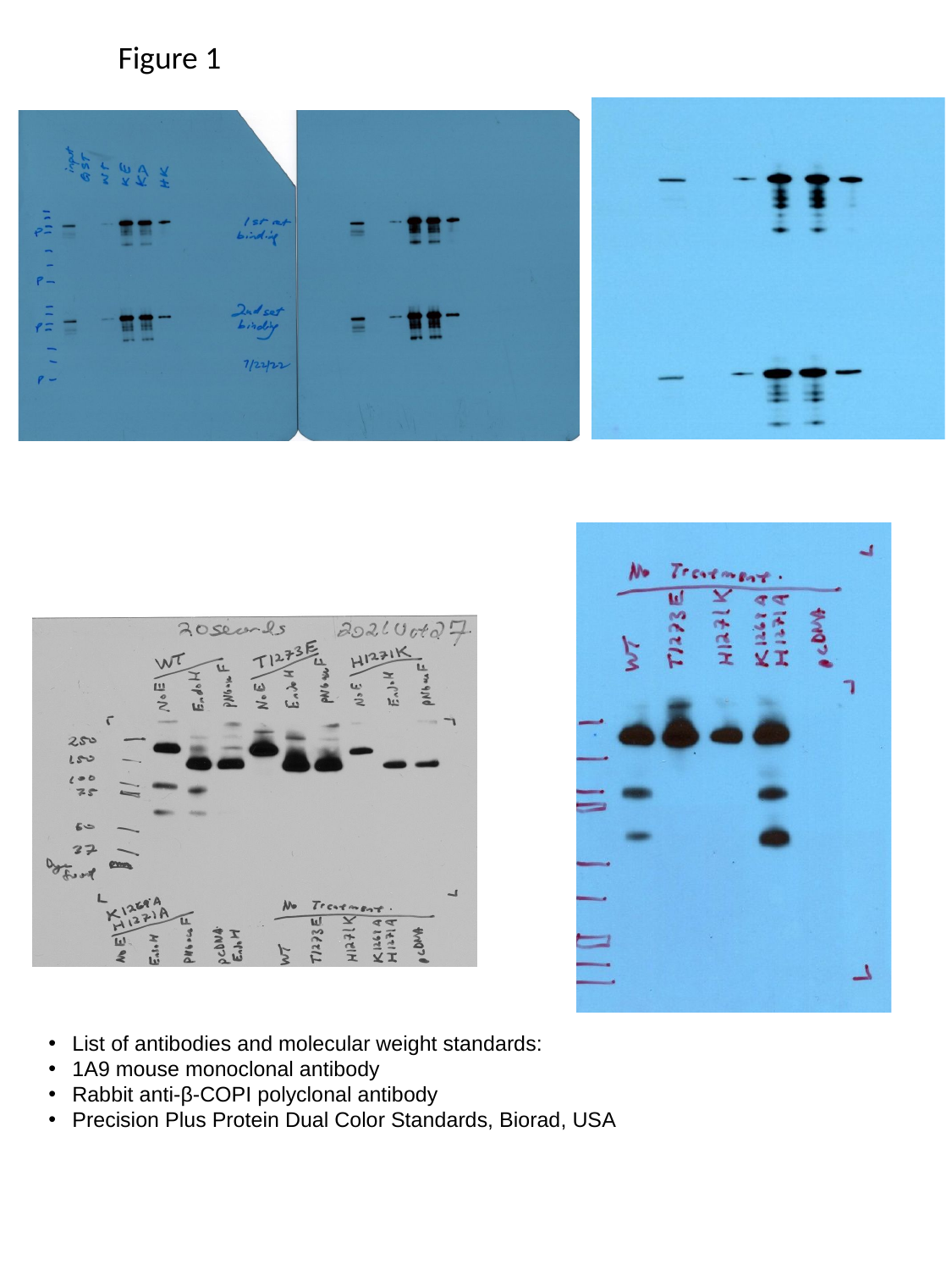

Figure 1
List of antibodies and molecular weight standards:
1A9 mouse monoclonal antibody
Rabbit anti-β-COPI polyclonal antibody
Precision Plus Protein Dual Color Standards, Biorad, USA

## Slide 2
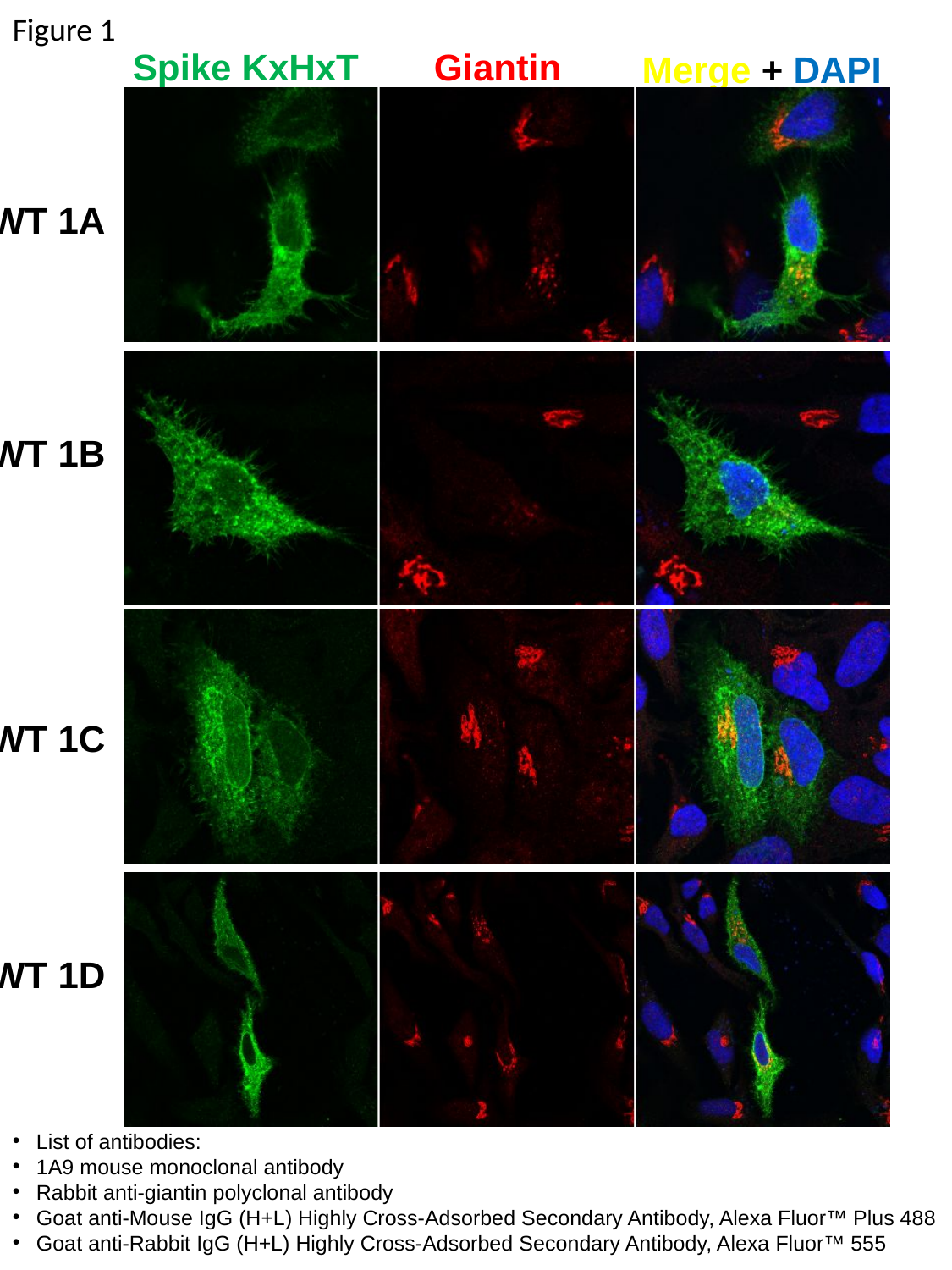

Figure 1
Spike KxHxT
Giantin
Merge + DAPI
WT 1A
WT 1B
WT 1C
WT 1D
List of antibodies:
1A9 mouse monoclonal antibody
Rabbit anti-giantin polyclonal antibody
Goat anti-Mouse IgG (H+L) Highly Cross-Adsorbed Secondary Antibody, Alexa Fluor™ Plus 488
Goat anti-Rabbit IgG (H+L) Highly Cross-Adsorbed Secondary Antibody, Alexa Fluor™ 555

## Slide 3
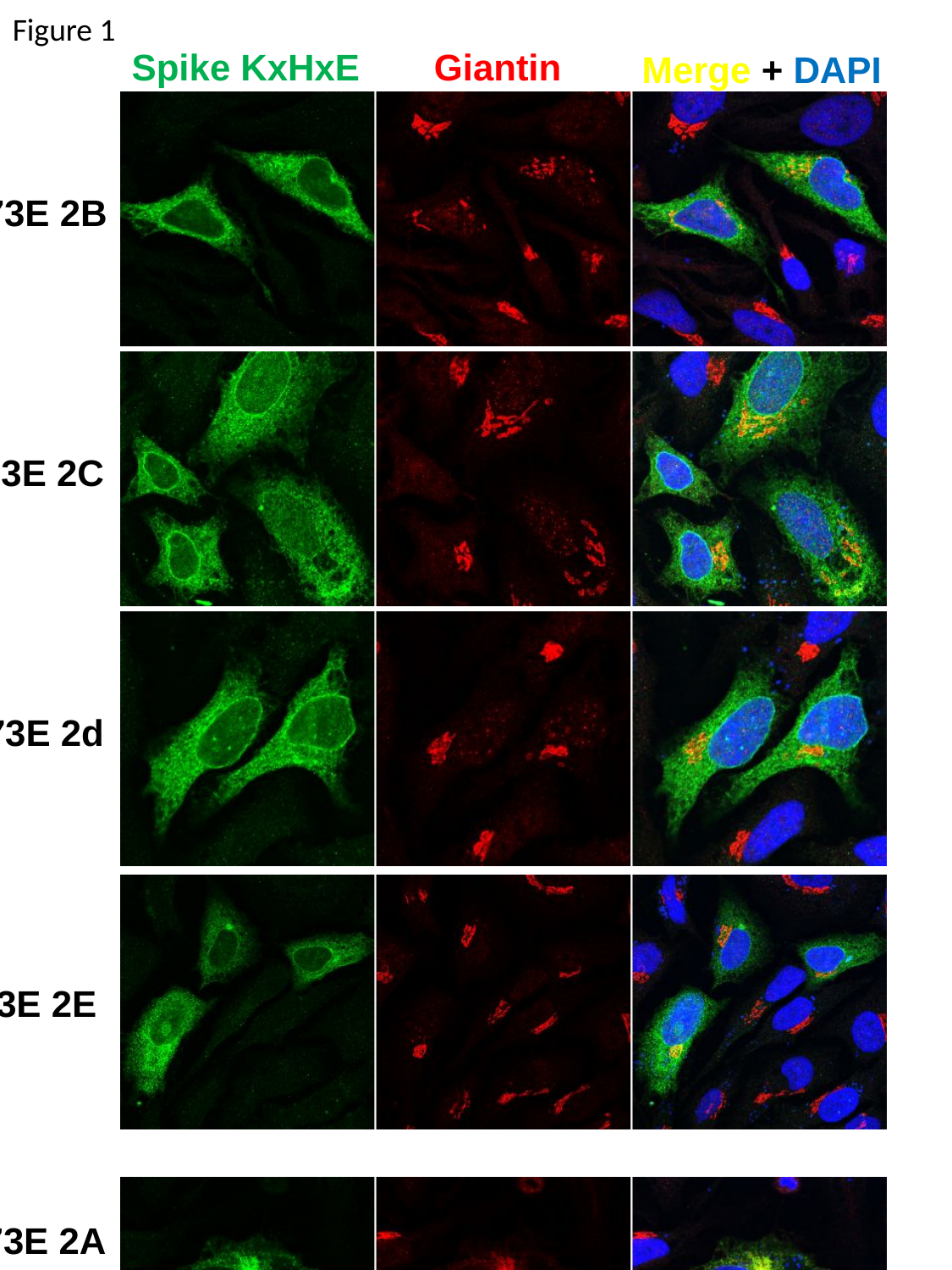

Figure 1
Spike KxHxE
Giantin
Merge + DAPI
Spike T1273E 3_ images used a different plasmid DNA prep (300 ng/uL) and different WT HeLa cells (50%) than T1273E 2_ images (460 ng/uL, 30% cells).
T1273E 2B
T1273E 2C
T1273E 2d
T1273E 2E
List of antibodies:
1A9 mouse monoclonal antibody
Rabbit anti-giantin polyclonal antibody
Goat anti-Mouse IgG (H+L) Highly Cross-Adsorbed Secondary Antibody, Alexa Fluor™ Plus 488
Goat anti-Rabbit IgG (H+L) Highly Cross-Adsorbed Secondary Antibody, Alexa Fluor™ 555
T1273E 2A

## Slide 4
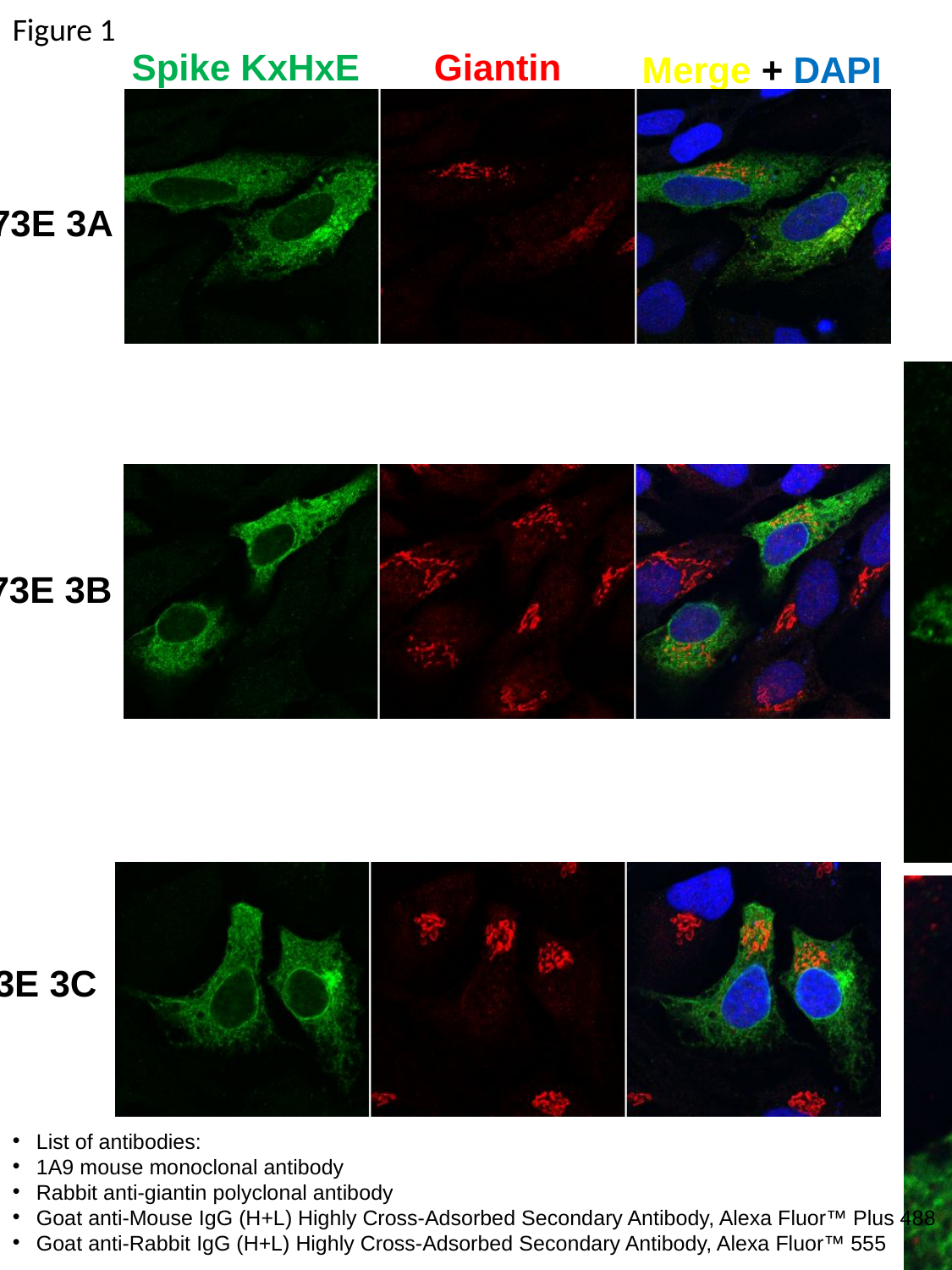

Figure 1
Spike KxHxE
Giantin
Merge + DAPI
T1273E 3_ used a different plasmid DNA prep (300 ng/uL) and different WT HeLa cells (50%) than T1273E 2_ images (460 ng/uL, 30% cells).
T1273E 3A
T1273E 3B
T1273E 3C
List of antibodies:
1A9 mouse monoclonal antibody
Rabbit anti-giantin polyclonal antibody
Goat anti-Mouse IgG (H+L) Highly Cross-Adsorbed Secondary Antibody, Alexa Fluor™ Plus 488
Goat anti-Rabbit IgG (H+L) Highly Cross-Adsorbed Secondary Antibody, Alexa Fluor™ 555

## Slide 5
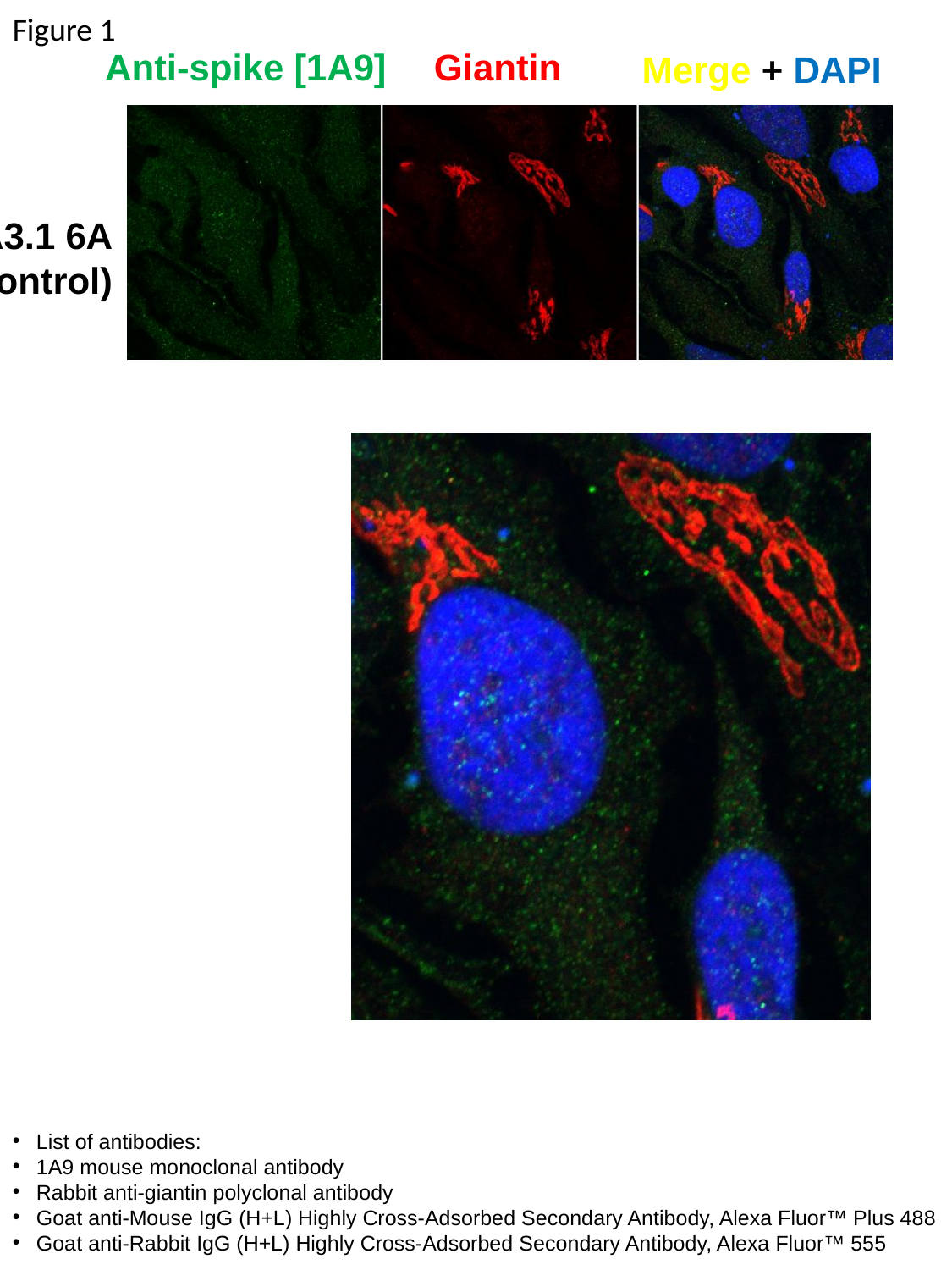

Figure 1
Anti-spike [1A9]
Giantin
Merge + DAPI
pcDNA3.1 6A
(plasmid control)
List of antibodies:
1A9 mouse monoclonal antibody
Rabbit anti-giantin polyclonal antibody
Goat anti-Mouse IgG (H+L) Highly Cross-Adsorbed Secondary Antibody, Alexa Fluor™ Plus 488
Goat anti-Rabbit IgG (H+L) Highly Cross-Adsorbed Secondary Antibody, Alexa Fluor™ 555

## Slide 6
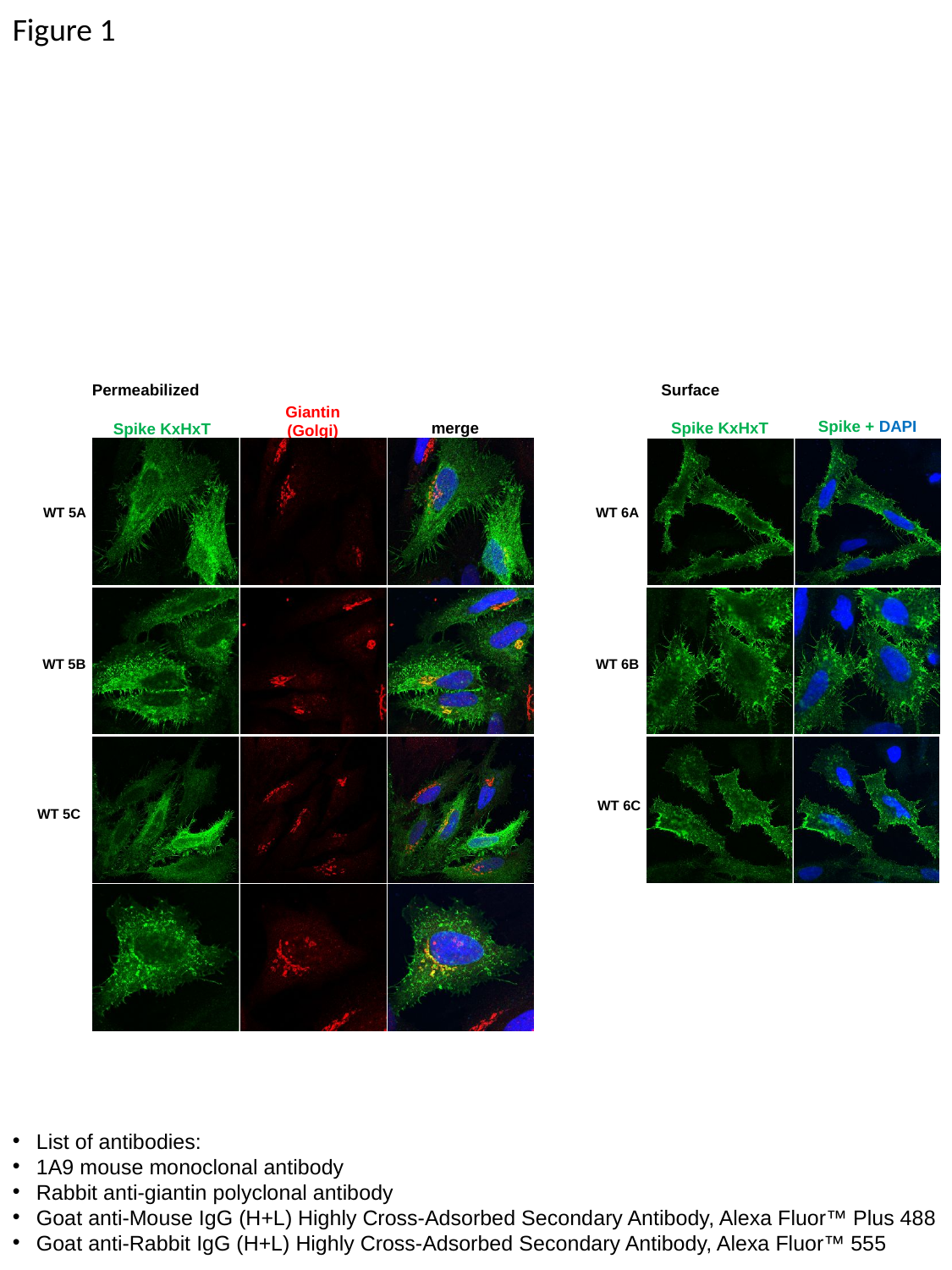

Figure 1
Permeabilized
Surface
Giantin
(Golgi)
Spike + DAPI
merge
Spike KxHxT
Spike KxHxT
WT 5A
WT 6A
WT 5B
WT 6B
WT 6C
WT 5C
List of antibodies:
1A9 mouse monoclonal antibody
Rabbit anti-giantin polyclonal antibody
Goat anti-Mouse IgG (H+L) Highly Cross-Adsorbed Secondary Antibody, Alexa Fluor™ Plus 488
Goat anti-Rabbit IgG (H+L) Highly Cross-Adsorbed Secondary Antibody, Alexa Fluor™ 555
WT 5E

## Slide 7
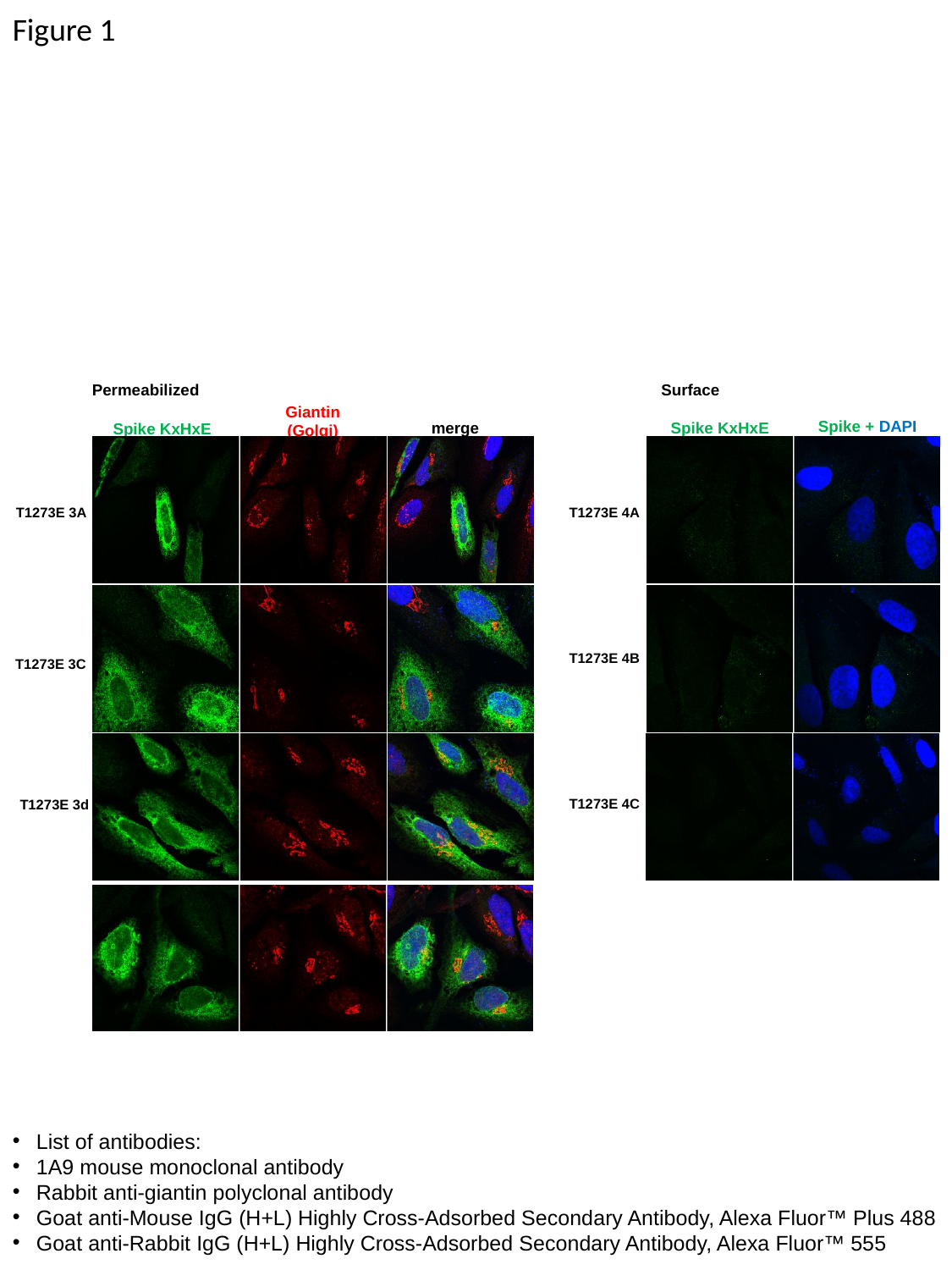

Figure 1
Permeabilized
Surface
Giantin
(Golgi)
Spike + DAPI
merge
Spike KxHxE
Spike KxHxE
T1273E 3A
T1273E 4A
T1273E 4B
T1273E 3C
T1273E 4C
T1273E 3d
List of antibodies:
1A9 mouse monoclonal antibody
Rabbit anti-giantin polyclonal antibody
Goat anti-Mouse IgG (H+L) Highly Cross-Adsorbed Secondary Antibody, Alexa Fluor™ Plus 488
Goat anti-Rabbit IgG (H+L) Highly Cross-Adsorbed Secondary Antibody, Alexa Fluor™ 555
T1273E 3E

## Slide 8
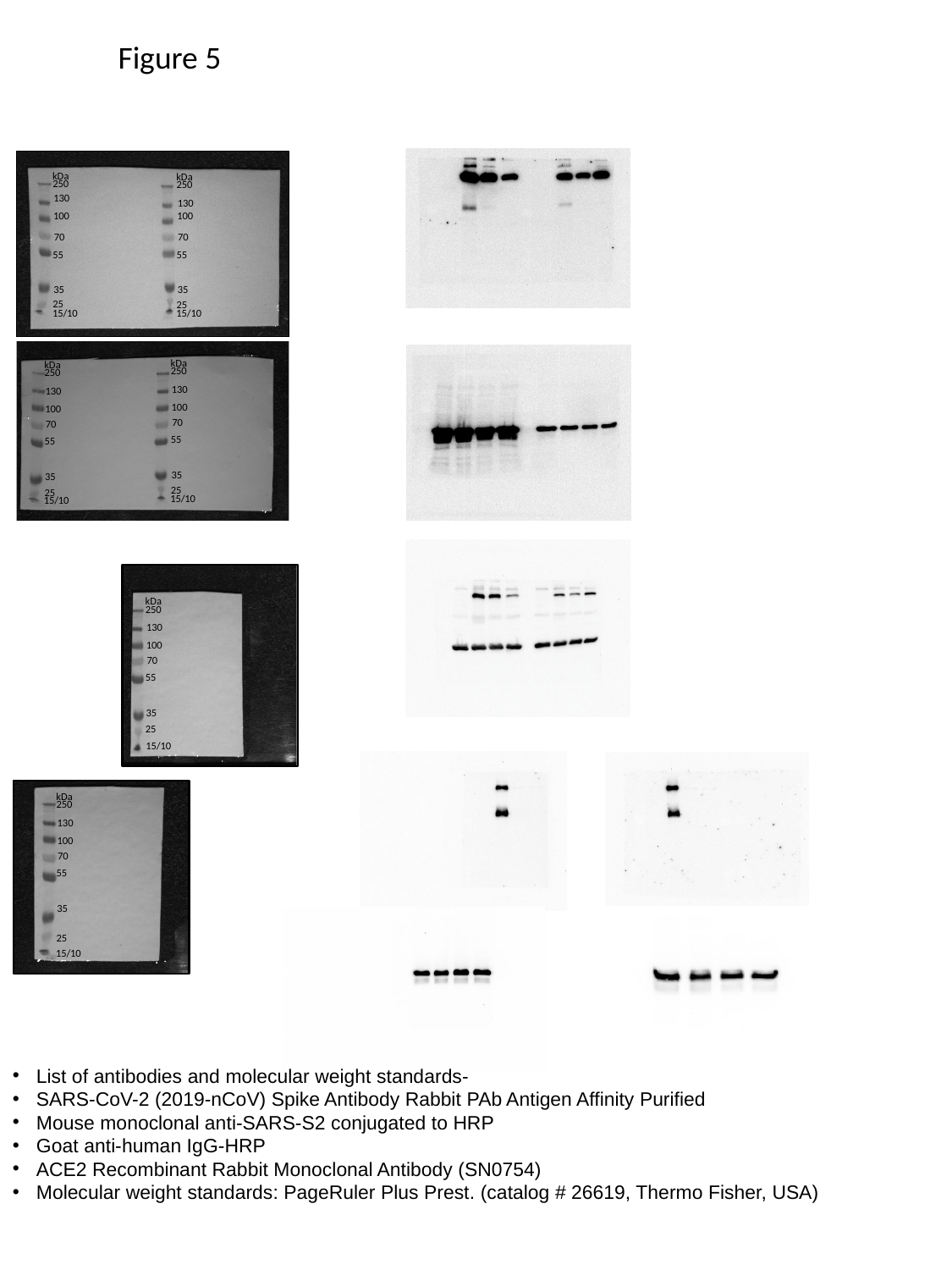

Figure 5
kDa
250
130
100
70
55
35
25
15/10
kDa
250
130
100
70
55
35
25
15/10
kDa
250
130
100
70
55
35
25
15/10
kDa
250
130
100
70
55
35
25
15/10
kDa
250
130
100
70
55
35
25
15/10
kDa
250
130
100
70
55
35
25
15/10
List of antibodies and molecular weight standards-
SARS-CoV-2 (2019-nCoV) Spike Antibody Rabbit PAb Antigen Affinity Purified
Mouse monoclonal anti-SARS-S2 conjugated to HRP
Goat anti-human IgG-HRP
ACE2 Recombinant Rabbit Monoclonal Antibody (SN0754)
Molecular weight standards: PageRuler Plus Prest. (catalog # 26619, Thermo Fisher, USA)

## Slide 9
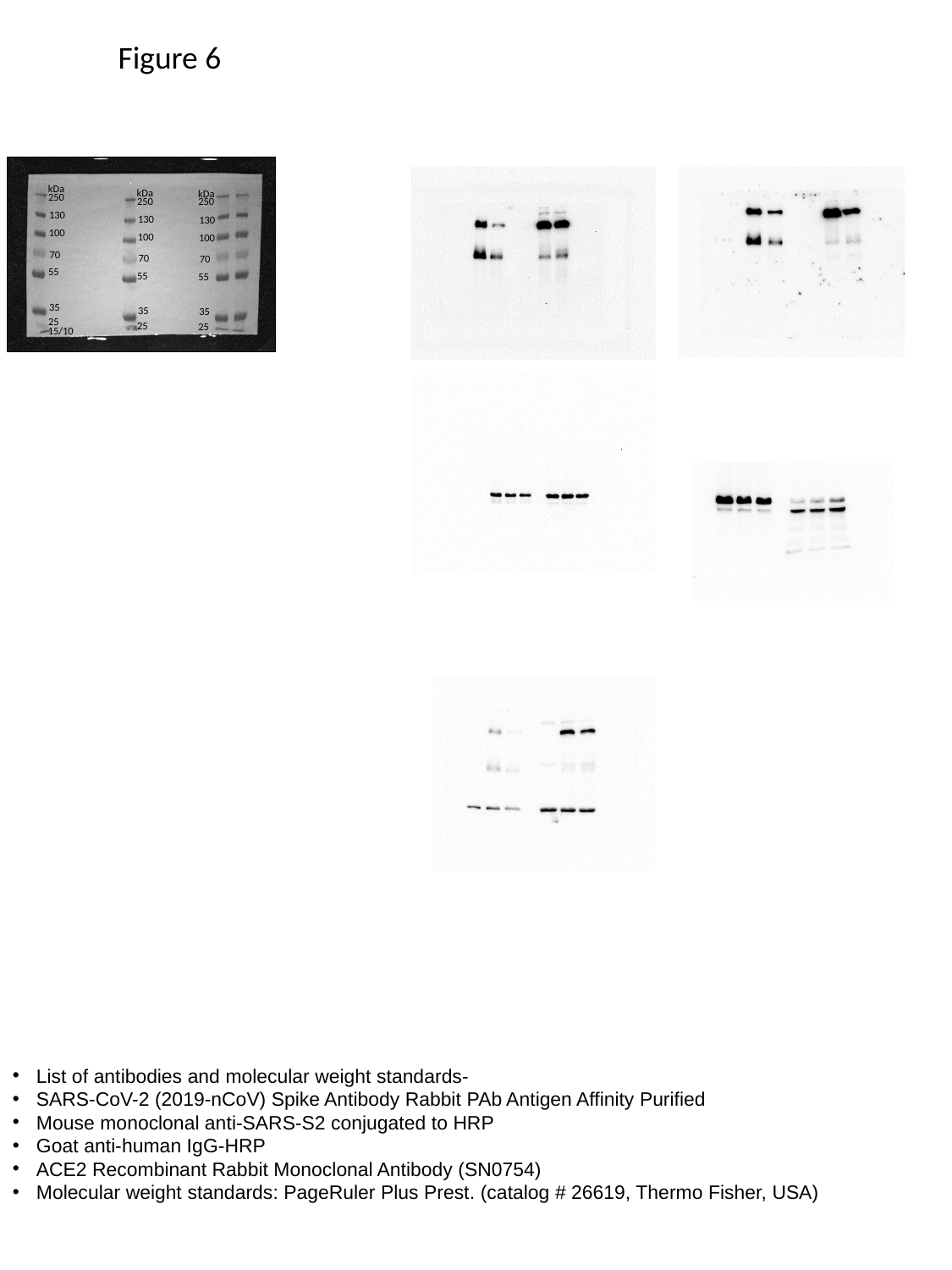

Figure 6
kDa
250
130
100
70
55
35
25
15/10
kDa
250
130
100
70
55
35
25
kDa
250
130
100
70
55
35
25
List of antibodies and molecular weight standards-
SARS-CoV-2 (2019-nCoV) Spike Antibody Rabbit PAb Antigen Affinity Purified
Mouse monoclonal anti-SARS-S2 conjugated to HRP
Goat anti-human IgG-HRP
ACE2 Recombinant Rabbit Monoclonal Antibody (SN0754)
Molecular weight standards: PageRuler Plus Prest. (catalog # 26619, Thermo Fisher, USA)
